# Two new lecanoroid lichen species from the forested wetlands of South Korea, with a key for Korean *Protoparmeliopsis* species

**DOI:** 10.3897/mycokeys.84.70798

**Published:** 2021-11-12

**Authors:** Beeyoung Gun Lee, Jae-Seoun Hur

**Affiliations:** 1 Baekdudaegan National Arboretum, Bonghwa 36209, South Korea Baekdudaegan National Arboretum Bonghwa Republic of Korea; 2 Korean Lichen Research Institute, Sunchon National University, Suncheon 57922, South Korea Sunchon National University Suncheon Republic of Korea

**Keywords:** Biodiversity, hygrophyte, Lecanoraceae, phylogeny, taxonomy

## Abstract

*Lecanoraparasymmicta* Lee & Hur and *Protoparmeliopsiscrystalliniformis* Lee & Hur are described as new lichen species to science from the forested wetlands in southern South Korea. Molecular analyses employing internal transcribed spacer (ITS) and mitochondrial small subunit (mtSSU) sequences strongly support the two lecanoroid species to be distinct in their genera. *Lecanoraparasymmicta* is included in the *Lecanorasymmicta* group. It is morphologically distinguished from *Lecanorasymmicta* (Ach.) Ach., its most similar species, by areolate-rimose thallus, blackish hypothallus, larger apothecia, absence of thalline excipulum from the beginning, narrower paraphyses, larger ascospores, smaller pycnoconidia, and the presence of placodiolic acid. The second new species *Protoparmeliopsiscrystalliniformis* is included in a clade with *Protoparmeliopsisbipruinosa* (Fink) S.Y. Kondr. and *P.nashii* (B.D. Ryan) S.Y. Kondr., differs from *Protoparmeliopsisertzii* Bungartz & Elix, its most morphologically similar species, by whitish thallus, flat to concave and paler disc, longer ascospores, thallus K+ yellow reaction, presence of atranorin and rhizocarpic acid, and the substrate preference to sandstone or basalt. A key is provided to assist in the identification of *Protoparmeliopsis* species in Korea.

## Introduction

As the genus *Lecanora* has been considered one of the largest genera in lichens, several infrageneric groups have been specifically or comprehensively studied in diverse aspects in morphology, chemistry and molecular phylogeny (Eigler 1969; Brodo 1984; Lumbsch 1995; Motyka 1995, 1996; Printzen 2001; Pérez-Ortega and Kantvilas 2010; Zhao et al. 2016; Bungartz et al. 2020). Main groups have been traditionally but informally recognized such as the *Lecanoradispersa* group, the *L.polytropa* group, the *L.rupicola* group, the *L.subfusca* group, the *L.symmicta* group, the *L.varia* group and the subgenus Placodium. The genera *Lecanoropsis*, *Myriolecis* and *Protoparmeliopsis* are originated from the *L.saligna*-, the *L.dispersa*-, and the *L.muralis*- groups, respectively (Śliwa et al. 2012; Zhao et al. 2016). Even a new genus *Sedelnikovaea* is differentiated from *Protoparmeliopsis*, one of the recently described genera (Kondratyuk et al. 2014). Other more groups have been defined such as the *L.carpinea*-, the *L.filamentosa*-, the *L.intumescens*-, the *L.subcarnea*- groups (Pérez-Ortega and Kantvilas 2010; Zhao et al. 2016), and the *L.pallida* group including the *L.subcarnea* group, the *L.marginata* group, and the *L.pinguis* group including a section in *Placodium* for the lecanoroid lichens of the Galapagos Islands (Bungartz et al. 2020).

Although there have been many groups classified as above, a few groups are proved more natural and homogenous and other groups are represented heterogenous without clarity in taxonomy (Zhao et al. 2016). The *Lecanoravaria* group s. lat. is one of the unclear groups, and some species in the group are classified into the *L.polytropa* group and other some species are nested into the *L.symmicta* group (Printzen and May 2002; Laundon 2003; Pérez-Ortega and Kantvilas 2018; Bungartz et al. 2020). The main difference between the latter two groups is that the *L.polytropa* group has the corticate apothecia becoming convex when mature and inhabits generally on well-lit acid rocks, but the *L.symmicta* group represents convex apothecia from the beginning and mainly inhabits barks or worked woods (Laundon 2003). Such an inconclusive group is in need of revision as other infrageneric groups have been revised (Śliwa and Flakus 2011).

Hue (1909) first reported the lecanoroid lichens from Korea by describing four new taxa in the genus *Lecanora*, *L.oreina* (Ach.) Ach., *L.hueana* Harm., L.hueanaf.microcarpa Hue, and *L.membranifera* Hue, although all the taxa are classified in other genera at present. Hur et al. (2005) arranged twelve species of *Lecanora* with specific references for each species reported from Korea. Moon (2013) listed twenty four species of *Lecanora* if we discard *L.fusanii* Hue (syn. *Caloplacafusanii* (Hue) Zahlbr.) and *L.vulnerata* Hue (syn. *Caloplacavulnerata* (Hue) Zahlbr.). Overall fifty two taxa had been recorded in Korea toward 2020 (Jeon et al. 2009; Joshi et al. 2009; Kondratyuk et al. 2013, Aptroot and Moon 2014, 2015; Kondratyuk et al. 2015, 2016a, 2016b, 2017; Lee and Hur 2020). *Protoparmeliopsis*, the lobate lecanoroid genus, was first referenced for Korea in 2007 and represented by *Protoparmeliopsismuralis* M. Choisy (Wei et al. 2007, sub *Lecanoramuralis* (Schreb.) Rabenh.). *Protoparmeliopsischejuensis* S.Y. Kondr. & Hur, *P.kopachevskae* S.Y. Kondr., Lőkös & Hur, *P.pseudogyrophorica* S.Y. Kondr., S.O. Oh & Hur, and *P.zerovii* S.Y. Kondr. were described or referenced from Korea during the 2010s (Kondratyuk et al. 2013, 2016a, 2017), and totally five species were recorded in the genus *Protoparmeliopsis* for the country, although *P.pseudogyrophorica* was later reclassified to *Sedelnikovaeapseudogyrophorica* (S. Y. Kondr., S.O. Oh & Hur) S. Y. Kondr. & Hur (Kondratyuk et al. 2019).

**Figure 1.**
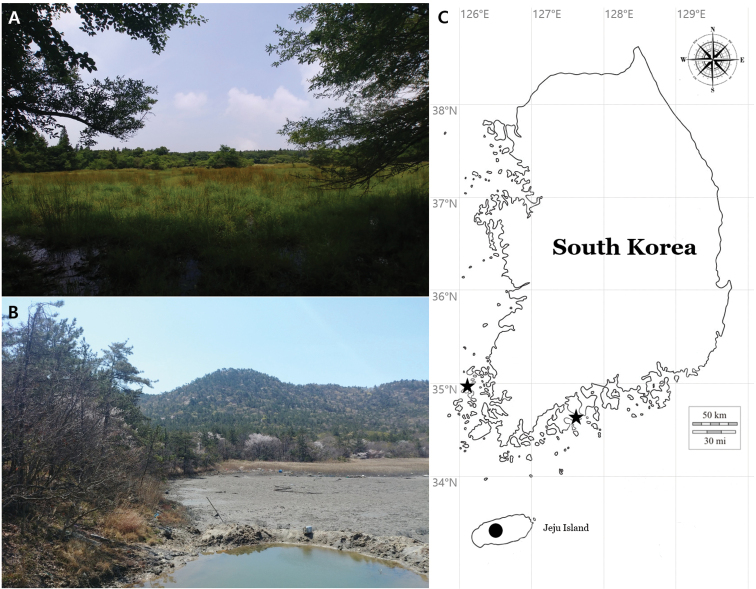
Specific collection sites for two new species **A** habitat/landscape of *Lecanoraparasymmicta***B** habitat/landscape of *Protoparmeliopsiscrystalliniformis***C** locations of *Lecanoraparasymmicta* (black circle) and *Protoparmeliopsiscrystalliniformis* (two black stars) on the map.

This study describes two new lichen-forming fungi species to science in the genera *Lecanora*, i.e., the *L.symmicta* group, and *Protoparmeliopsis*. Field surveys for the lichen biodiversity in the forested wetlands of southern South Korea were accomplished during the summer of 2020, and a few dozen specimens were collected in the wetland forests nearby seashore or in islands (Fig. 1). The collected specimens were comprehensively analyzed in ecology, morphology, chemistry and molecular phylogeny and did not correspond to any previously known species. We describe them as two new species, *Lecanoraparasymmicta* and *Protoparmeliopsiscrystalliniformis*, and these discoveries contributes to the taxonomy of the lecanoroid lichens of Korea by listing overall fifty three taxa of *Lecanora* and six taxa of *Protoparmeliopsis*. The specimens are deposited in the herbarium of the Baekdudaegan National Arboretum (KBA, the herbarium acronym in the Index Herbariorum), South Korea.

## Materials and methods

### Morphological and chemical analyses

Hand sections were prepared manually with a razor blade under a stereomicroscope (Olympus optical SZ51; Olympus, Tokyo, Japan), scrutinized under a compound microscope (Nikon Eclipse E400; Nikon, Tokyo, Japan) and pictured using a software program (NIS-Elements D; Nikon, Tokyo, Japan) and a DS-Fi3 camera (Nikon, Tokyo, Japan) mounted on a Nikon Eclipse Ni-U microscope (Nikon, Tokyo, Japan). The ascospores were examined at 1000× magnification in water. The length and width of the ascospores were measured and the range of spore sizes was shown with average, standard deviation (SD), length-to-width ratio, and number of measured spores. Thin-layer chromatography (TLC) was performed using solvent systems A and C according to standard methods (Orange et al. 2001).

### Isolation, DNA extraction, amplification, and sequencing

Hand-cut sections of ten to twenty ascomata per collected specimen were prepared for DNA isolation and DNA was extracted with a NucleoSpin Plant II Kit in line with the manufacturer’s instructions (Macherey-Nagel, Düren, Germany). PCR amplification for the internal transcribed spacer region (ITS1-5.8S-ITS2 rDNA), the mitochondrial small subunit, and the nuclear large subunit ribosomal RNA genes was achieved using Bioneer’s AccuPower PCR Premix (Bioneer, Daejeon, Korea) in 20-μl tubes with 16 μl of distilled water, 2 μl of DNA extracts and 2 μl of primers ITS5 and ITS4 (White et al. 1990), mrSSU1 and mrSSU3R (Zoller et al. 1999) or LR0R and LR5 (Rehner and Samuels 1994). The PCR thermal cycling parameters used were 95 °C (15 sec), followed by 35 cycles of 95 °C (45 sec), 54 °C (45 sec), and 72 °C (1 min), and a final extension at 72 °C (7 min) based on Ekman (2001). The annealing temperature was occasionally altered by ±1 degree in order to get a better result. PCR purification and DNA sequencing were accomplished by the genomic research company Macrogen (Seoul, Korea).

### Phylogenetic analyses

All ITS and mtSSU sequences were aligned and edited manually using ClustalW in Bioedit V7.2.6.1 (Hall 1999). All missing and ambiguously aligned data and parsimony-uninformative positions were removed and only parsimony-informative regions were finally analyzed in MEGA X (Stecher et al. 2020). The final alignment comprised 1462 (ITS) and 1058 (mtSSU) columns for *Lecanora*. In them, variable regions were 171 (ITS) and 117 (mtSSU). The phylogenetically informative regions were 440 (ITS) and 152 (mtSSU). The final alignment for *Protoparmeliopsis* comprised 945 (ITS) and 985 (mtSSU) columns. In them, variable regions were 214 (ITS) and 53 (mtSSU). Finally, the phylogenetically informative regions were 268 (ITS) and 134 (mtSSU). Phylogenetic trees with bootstrap values were obtained in RAxML GUI 2.0 beta (Edler et al. 2019) using the Maximum Likelihood method with a rapid bootstrap with 1000 bootstrap replications and GTR GAMMA for the substitution matrix. The posterior probabilities were obtained in BEAST 2.6.4 (Bouckaert et al. 2019) using the GTR 123141 (ITS for *Lecanora*), the GTR 121321 (mtSSU for *Lecanora*), the HKY (Hasegawa-Kishino-Yano) (ITS for *Protoparmeliopsis*), and the GTR 123123 (mtSSU for *Protoparmeliopsis*) models, as the appropriate models of nucleotide substitution produced by the Bayesian model averaging methods with bModelTest (Bouckaert and Drummond 2017), empirical base frequencies, gamma for the site heterogeneity model, four categories for gamma, and a 10,000,000 Markov chain Monte Carlo chain length with a 10,000-echo state screening and 1000 log parameters. Then, a consensus tree was constructed in TreeAnnotator 2.6.4 (Bouckaert et al. 2019) with no discard of burnin, no posterior probability limit, a maximum clade credibility tree for the target tree type, and median node heights. All trees were displayed in FigTree 1.4.2 (Rambaut 2014) and edited in Microsoft Paint. The bootstrapping and Bayesian analyses were repeated three times for the result consistency and no significant differences were shown for the tree shapes and branch values. The phylogenetic trees and DNA sequence alignments are deposited in TreeBASE under the study ID 28189. Overall analyses in the materials and methods were accomplished based on Lee and Hur (2020).

## Results and discussion

### Phylogenetic analyses

**Figure 2.**
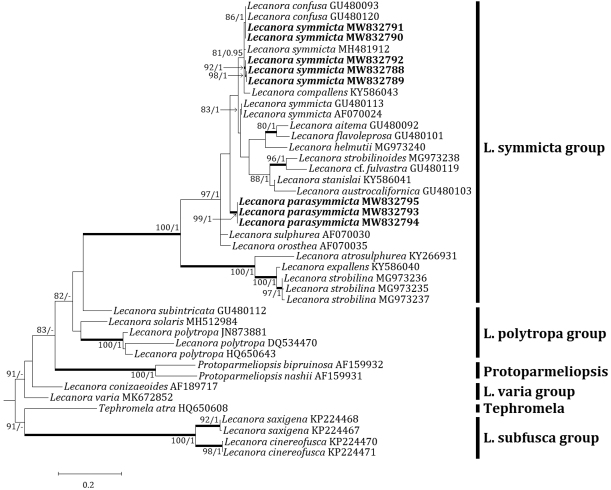
Phylogenetic relationships amongst available species in the *Lecanorasymmicta* group based on a Maximum Likelihood analysis of the dataset of ITS sequences. The tree was rooted with five sequences of the *Lecanorasubfusca* group and *Tephromela*. Maximum Likelihood bootstrap values ≥ 70% and posterior probabilities ≥ 95% are shown above internal branches. Branches with bootstrap values ≥ 90% are shown in bold. The new sequences of *Lecanoraparasymmicta* and *Lecanorasymmicta* produced from this study are presented in bold, and all species names are followed by the GenBank accession numbers. Reference Table 1 provides the species related to the specific GenBank accession numbers and voucher information.

**Figure 3.**
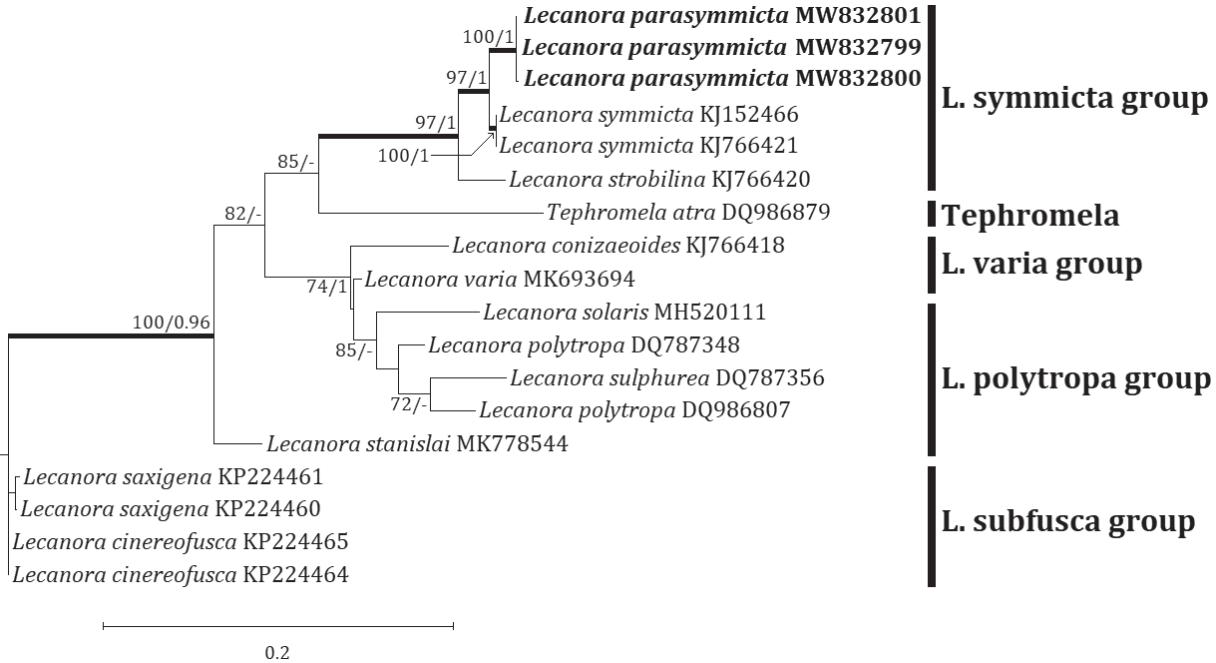
Phylogenetic relationships amongst available species in the *Lecanorasymmicta* group based on a Maximum Likelihood analysis of the dataset of the mitochondrial small subunit (mtSSU) sequences. The tree was rooted with four sequences of the *Lecanorasubfusca* group. Maximum Likelihood bootstrap values ≥ 70% and posterior probabilities ≥ 95% are shown above internal branches. Branches with bootstrap values ≥ 90% are shown in bold. The new species *Lecanoraparasymmicta* is presented in bold, and all species names are followed by the GenBank accession numbers. Reference Table 1 provides the species related to the specific GenBank accession numbers and voucher information.

**Figure 4.**
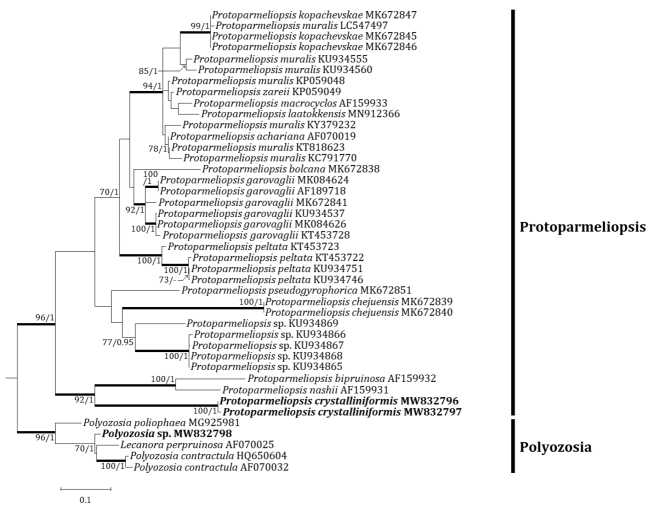
Phylogenetic relationships amongst available species in the genus *Protoparmeliopsis* based on a Maximum Likelihood analysis of the dataset of ITS sequences. The tree was rooted with five sequences of the genus *Polyozosia*. Maximum Likelihood bootstrap values ≥ 70% and posterior probabilities ≥ 95% are shown above internal branches. Branches with bootstrap values ≥ 90% are shown in bold. The new species *Protoparmeliopsiscrystalliniformis* is presented in bold, and all species names are followed by the GenBank accession numbers. Reference Table 1 provides the species related to the specific GenBank accession numbers and voucher information.

**Figure 5.**
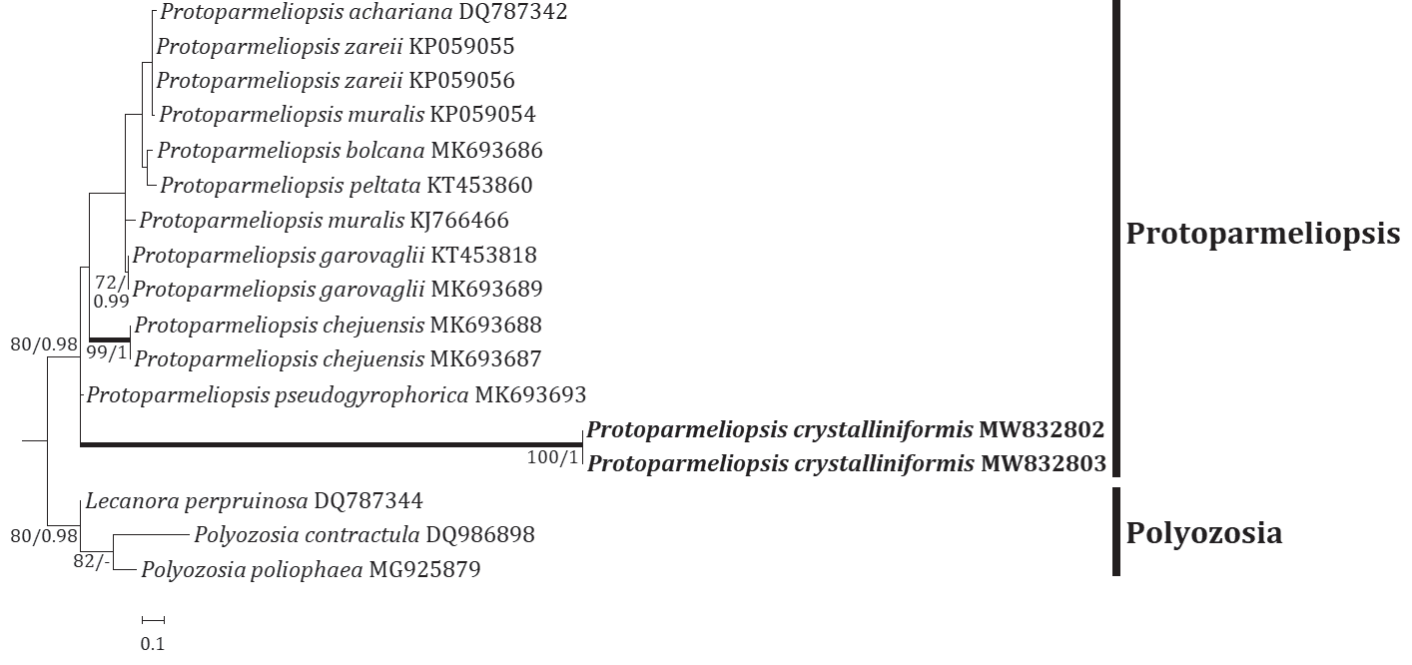
Phylogenetic relationships amongst available species in the genus *Protoparmeliopsis* based on a Maximum Likelihood analysis of the dataset of the mitochondrial small subunit (mtSSU) sequences. The tree was rooted with three sequences of the genus *Polyozosia*. Maximum Likelihood bootstrap values ≥ 70% and posterior probabilities ≥ 95% are shown above internal branches. Branches with bootstrap values ≥ 90% are shown in bold. The new species *Protoparmeliopsiscrystalliniformis* is presented in bold, and all species names are followed by the GenBank accession numbers. Reference Table 1 provides the species related to the specific GenBank accession numbers and voucher information.

Four independent phylogenetic trees for the genera *Lecanora* and *Protoparmeliopsis* were produced from 117 sequences (71 for ITS, and 30 for mtSSU) from GenBank and, 16 new sequences (11 for ITS and 5 for mtSSU) from the new and compared species (Table 1). *Lecanoraparasymmicta*, one of the new species, is positioned in the *L.symmicta* group in both ITS and mtSSU trees. The ITS tree illustrates that the new species is located in its own clade without any species close to it. *Lecanorasymmicta*, the most similar species, is positioned in a clade with *L.confusa* Almb. and *L.compallens* Herk & Aptroot, situated far from the new species (Fig. 2). The mtSSU tree shows that the new species is located in a clade with *L.symmicta* and *L.strobilina* Ach., represented by a bootstrap value of 100 and a posterior probability of 1.0 for the branch (Fig. 3). The second new species, *Protoparmeliopsiscrystalliniformis*, was positioned in *Protoparmeliopsis* in both ITS and mtSSU trees. The ITS tree explains that the new species is located in a clade with *P.bipruinosa* (Fink) S.Y. Kondr. and *P.nashii* (B.D. Ryan) S.Y. Kondr., represented by a bootstrap value of 92 and a posterior probability of 1.0 for the branch (Fig. 4). The mtSSU tree shows that *P.crystalliniformis* is located in its own clade (Fig. 5). The phylogenetic analyses, and according to the included taxa, did not indicate any such species to the two new proposed in *Lecanora* and *Protoparmeliopsis*.

**Table 1.** Species list and DNA sequence information employed for phylogenetic analysis.

No.	Species	ID (ITS)	ID (mtSSU)	Voucher
1	*Lecanoraaitema*	GU480092		SPO1
2	*Lecanoraatrosulphurea*	KY266931		O-L-195558
3	*Lecanoraaustrocalifornica*	GU480103		SPO2
4	*Lecanoracinereofusca*	KP224470	KP224465	Lendemer 34944 (NY)
5	*Lecanoracinereofusca*	KP224471	KP224464	Lendemer 35007 (NY)
6	*Lecanoracompallens*	KY586043		JM6948
7	*Lecanoraconfusa*	GU480093		SPO10
8	*Lecanoraconfusa*	GU480120		SPO9
9	*Lecanoraconizaeoides*	AF189717		U229
10	*Lecanoraconizaeoides*		KJ766418	AFTOL-ID 1858
11	*Lecanoraexpallens*	KY586040		UGDA-L17316
12	*Lecanoraflavoleprosa*	GU480101		SPO18
13	Lecanoracf.fulvastra	GU480119		SPO8
14	*Lecanorahelmutii*	MG973240		MA:Lichen:19506
15	*Lecanoraorosthea*	AF070035		U244
**16**	***Lecanoraparasymmicta***	**MW832793**	**MW832799**	**BDNA-L-0001218**
**17**	***Lecanoraparasymmicta***	**MW832794**	**MW832800**	**BDNA-L-0001220**
**18**	***Lecanoraparasymmicta***	**MW832795**	**MW832801**	**BDNA-L-0001235**
19	*Lecanoraperpruinosa*	AF070025		U176
20	*Lecanoraperpruinosa*		DQ787344	U506
21	*Lecanorapolytropa*	DQ534470		Hur ANT050752
22	*Lecanorapolytropa*	HQ650643	DQ986807	AFTOL-ID 1798
23	*Lecanorapolytropa*	JN873881		U.C. Riverside 47815UCR1
24	*Lecanorapolytropa*		DQ787348	U520
25	*Lecanorasaxigena*	KP224467	KP224460	Lendemer 25832 (NY)
26	*Lecanorasaxigena*	KP224468	KP224461	Lendemer 33186 (NY)
27	*Lecanorasolaris*	MH512984		LYF14–69
28	*Lecanorasolaris*		MH520111	ED (14336) & LY
29	*Lecanorastanislai*	KY586041		UGDA-L17244
30	*Lecanorastanislai*		MK778544	J. Malicek 10367
31	*Lecanorastrobilina*	MG973235		MA:Lichen:19510
32	*Lecanorastrobilina*	MG973236		MA:Lichen:19511
33	*Lecanorastrobilina*	MG973237		MA:Lichen:19509
34	*Lecanorastrobilina*		KJ766420	DUKE:M. Kukwa 4761
35	*Lecanorastrobilinoides*	MG973238		MA:Lichen:19507
36	*Lecanorasubintricata*	GU480112		SPO28
37	*Lecanorasulphurea*	AF070030		U212
38	*Lecanorasulphurea*		DQ787356	U508
39	*Lecanorasymmicta*	AF070024		U205
40	*Lecanorasymmicta*	GU480113		SPO29
41	*Lecanorasymmicta*	MH481912		O-L-209831
**42**	***Lecanorasymmicta***	**MW832788**		**BDNA-L-0000547**
**43**	***Lecanorasymmicta***	**MW832789**		**BDNA-L-0000548(br)**
**44**	***Lecanorasymmicta***	**MW832790**		**BDNA-L-0000548(yel)**
**45**	***Lecanorasymmicta***	**MW832791**		**BDNA-L-0000551**
**46**	***Lecanorasymmicta***	**MW832792**		**BDNA-L-0000642**
47	*Lecanorasymmicta*		KJ766421	EGR:K. Molnar 23-08-2005/B
48	*Lecanorasymmicta*		KJ152466	C. Printzen 9999a (FR)
49	*Lecanoravaria*	MK672852	MK693694	Kondratyuk S. 21325 (KW-L)
50	*Polyozosiacontractula*	AF070032		U236
51	*Polyozosiacontractula*	HQ650604	DQ986898	AFTOL-ID 877
52	*Polyozosiapoliophaea*	MG925981	MG925879	O:L 200460
**53**	***Polyozosia* sp.**	**MW832798**		**BDNA-L-0001105**
54	*Protoparmeliopsisachariana*	AF070019		U155
55	*Protoparmeliopsisachariana*		DQ787342	U525
56	*Protoparmeliopsisbipruinosa*	AF159932		U354
57	*Protoparmeliopsisbolcana*	MK672838	MK693686	Kondratyuk S. 20309 (KW-L)
58	*Protoparmeliopsischejuensis*	MK672839	MK693687	KoLRI 022622
59	*Protoparmeliopsischejuensis*	MK672840	MK693688	KoLRI 022618
**60**	***Protoparmeliopsiscrystalliniformis***	**MW832796**	**MW832802**	**BDNA-L-0000298**
**61**	***Protoparmeliopsiscrystalliniformis***	**MW832797**	**MW832803**	**BDNA-L-0000349**
62	*Protoparmeliopsisgarovaglii*	AF189718		M107
63	*Protoparmeliopsisgarovaglii*	KT453728	KT453818	Leavitt 089 (BRY-C)
64	*Protoparmeliopsisgarovaglii*	KU934537		Leavitt 199 (BRY-C)
65	*Protoparmeliopsisgarovaglii*	MK084624		Szczepanska 1240
66	*Protoparmeliopsisgarovaglii*	MK084626		Flakus 21175
67	*Protoparmeliopsisgarovaglii*	MK672841	MK693689	M. Haji Moniri (KW-L)
68	*Protoparmeliopsiskopachevskae*	MK672845		KoLRI 040224
69	*Protoparmeliopsiskopachevskae*	MK672846		KoLRI 040267
70	*Protoparmeliopsiskopachevskae*	MK672847		KoLRI 040276
71	*Protoparmeliopsislaatokaensis*	MN912366		20132508
72	*Protoparmeliopsismacrocyclos*	AF159933		U273
73	*Protoparmeliopsismuralis*	KC791770		BGK247
74	*Protoparmeliopsismuralis*	KP059048	KP059054	SK 765
75	*Protoparmeliopsismuralis*	KT818623		DNA 9890 (F)
76	*Protoparmeliopsismuralis*	KU934555		Leavitt 146 (BRY-C)
77	*Protoparmeliopsismuralis*	KU934560		Vondrak 106b (PRA)
78	*Protoparmeliopsismuralis*	KY379232		BGK257
79	*Protoparmeliopsismuralis*	LC547497		CBM:FL-41434
80	*Protoparmeliopsismuralis*		KJ766466	EGR:K. Molnar U0501/AO
81	*Protoparmeliopsisnashii*	AF159931		U253
82	*Protoparmeliopsispeltata*	KT453722	KT453860	
83	*Protoparmeliopsispeltata*	KT453723		MS014622
84	*Protoparmeliopsispeltata*	KU934746		Kaz 13085pelt
85	*Protoparmeliopsispeltata*	KU934751		Vondrak V127 (PRA)
86	*Protoparmeliopsispseudogyrophorica*	MK672851	MK693693	KoLRI 016651
87	*Protoparmeliopsiszareii*	KP059049	KP059055	SK 480
88	*Protoparmeliopsiszareii*		KP059056	SK 481
89	*Protoparmeliopsis* sp.	KU934865		Vondrak 9980 (PRA)
90	*Protoparmeliopsis* sp.	KU934866		Vondrak 10043 (PRA)
91	*Protoparmeliopsis* sp.	KU934867		Vondrak 10044 (PRA)
92	*Protoparmeliopsis* sp.	KU934868		Vondrak 10055 (PRA)
93	*Protoparmeliopsis* sp.	KU934869		Vondrak 9992 (PRA)
94	*Tephromelaatra*	HQ650608	DQ986879	AFTOL-ID 1373
**Overall**		**82**	**35**	

DNA sequences which were generated in this study, in bold the new species *Lecanoraparasymmicta* and *Protoparmeliopsiscrystalliniformis* and newly generated sequences of *Lecanorasymmicta* and *Polyozosia* sp. specimens. All others were obtained from GenBank. The species names are followed by GenBank accession numbers and voucher information. ITS, internal transcribed spacer; mtSSU, mitochondrial small subunit; Voucher, voucher information.

### Taxonomy

#### Lecanora parasymmicta

Taxon classificationFungiLecanoralesLecanoraceae

B.G. Lee & J.-S. Hur sp. nov.

C25D7BF4-D660-5B17-B2D5-90A04CF0E69D

839182

Fig. 6

##### Diagnosis.

*Lecanoraparasymmicta* differs from *L.symmicta*, the most similar species, by its areolate-rimose thallus (vs. areolate to leprose thallus), blackish hypothallus (vs. hypothallus indistinct), larger apothecia (up to 1.7 mm diam. vs. up to 1 mm diam.), absence of thalline excipulum from the beginning (vs. presence of thalline excipulum when young at least), narrower paraphyses (1–1.5 μm vs. 2–2.5 μm), larger ascospores (11–18 × 4–7 μm vs. 9–15.5 × 4–5 μm), smaller pycnoconidia (12–21 × 0.5–0.8 μm vs. 18–25 × 0.5–1.0 μm), chemical reactions (thallus K± slightly yellow, C–, KC– and UV– vs. K–, C± orange, KC± slightly yellow, UV+ dull orange), and the presence of placodiolic acid (vs. presence of arthothelin and ±thiophanic acid).

##### Type.

South Korea, Jeju Island, Aewol-eup, Gwangnyeongri/bongseongri, Mt. Halla, a forested wetland, 33°21.85'N, 126°26.91'E, 980 m alt., on bark of *Maackiafauriei* (H. Lév.) Takeda, 08 July 2020, B.G.Lee & H.J.Lee 2020-001020, with *Graphisscripta* (L.) Ach. (holotype: BDNA-L-0001220; GenBank MW832794 for ITS and MW832800 for mtSSU); same locality, on bark of *Malussieboldii* (Regel) Rehder, 08 July 2020, B.G.Lee & H.J.Lee 2020-001018, (paratype: BDNA-L-0001218; GenBank MW832793 for ITS and MW832799 for mtSSU); same locality, on bark of *Malussieboldii*, 08 July 2020, B.G.Lee & H.J.Lee 2020-001019, with Phaeographisaff.inusta (paratype: BDNA-L-0001219); same locality, on bark of *Maackiafauriei*, 08 July 2020, B.G.Lee & H.J.Lee 2020-001026, (paratype: BDNA-L-0001226); same locality, on bark of *Maackiafauriei*, 08 July 2020, B.G.Lee & H.J.Lee 2020-001035, with *Lecanoramegalocheila* (Hue) H. Miyaw. (paratype: BDNA-L-0001235; GenBank MW832795 for ITS and MW832801 for mtSSU); same locality, on bark of *Ligustrumobtusifolium* Siebold & Zucc., 08 July 2020, B.G.Lee & H.J.Lee 2020-001036, with *Graphisscripta* (paratype: BDNA-L-0001236).

##### Description.

Thallus corticolous, crustose, areolate to rimose but not leprose, light olivish gray to light gray, margin determinate, not pruinose, 60–200 μm thick; cortex hyaline, 5–10 μm thick; medulla often intermixed with algae and even with bark layer, small crystals in cortex or between algae, dissolving in K; photobiont coccoid, cells globose to ellipsoid, 5–15 μm. Hypothallus blackish.

Apothecia abundant, rounded, often contiguous or even coalescent, emerging on the surface of thallus and sessile when mature but margin generally attached to thallus surface, constricted at the base, 0.3–1.7 mm diam. Disc flat in the beginning and soon convex, smooth or becoming rugose by apothecia adjoining, not pruinose or slightly pruinose, pale yellow in the beginning and slightly darker when mature, sometimes with dark spots (algae), 180–400 μm thick; biatorine. Thalline excipulum absent from the beginning, proper excipulum present and sometimes slightly paler than disc, more distinctive when young, hyaline but yellowish brown to pale brown at periphery with granules which dissolving in K, periphery color same to epihymenium, ca. 90 μm wide laterally and 70–80 μm wide at periphery, disappearing to the base. Epihymenium yellowish brown to pale brown, granular, dissolving in K, 10–20 μm high. Hymenium hyaline, 70–90μm high. Subhymenium hyaline, 30–50 μm high. Hypothecium hyaline, prosoplectenchymatous (irregular), 50–60 μm high. Crystals and oil droplets absent in apothecial section. Paraphyses septate, anastomosing, 1–1.5 μm wide, simple or branched at tips, tips not swollen or slightly swollen, not pigmented, epihymenium pigmented by granules, not by paraphysial tips, ca. 1.5 μm wide. Asci clavate, 8-spored, 50–60 × 13–21 μm (n = 7). Ascospores constantly simple but rarely 1-septate, coarsely biseriate or irregularly arranged, 11–18 × 4–7 μm (mean = 13.8 × 5.8 μm; SD = 1.62(L), 0.63(W); L/W ratio 1.8–4.0, ratio mean = 2.4, ratio SD = 0.3; n = 105). Pycnidia immersed, ostiolar region slightly projected with a thalline excipulum, round to irregularly asymmetric, brown to black, 220 × 180 μm. Pycnoconidia thread-like, generally curved, 12–21 × 0.5–0.8 μm.

**Figure 6.**
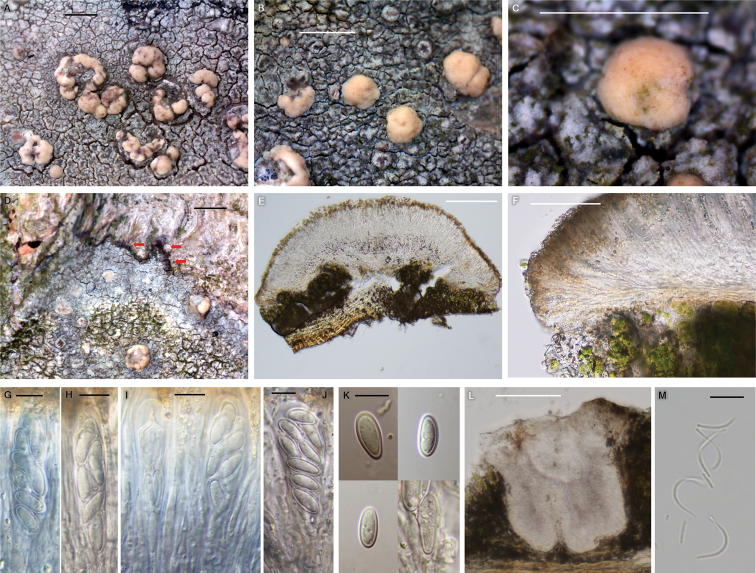
*Lecanoraparasymmicta* morphology (BDNA-L-0001235, paratype in **A** BDNA-L-0001220, holotype in **B–M**) **A–C** habitus and apothecia, thalline margin of apothecia consistently absent from the beginning **D** blackish hypothallus (red arrows) **E** apothecia in vertical section **F** biatorine apothecia without thalline margin **G–J** clavate asci with eight spores **K** ascospores constantly simple but rarely 1-septate **L** immersed pycnidia **M** thread-like, curved pycnoconidia. Scale bars: 1 mm (**A–D**); 200 μm (**E**); 50 μm (**F**); 10 μm (**G–K**); 100 μm (**L**); 10 μm (**M**).

##### Chemistry.

Thallus K– or K+ slightly yellowish, KC–, C–, Pd–. Hymenium, epihymenium and ascus tholus I+ blue. UV–. Usnic acid, zeorin, and placodiolic acid were detected by TLC.

**Table 2.** Comparison of the new species with close species in the *Lecanorasymmicta* group.

Species	*L.parasymmicta*	*L.aitema*	*L.confusa*	*L.strobilina*	*L.symmicta*
Thallus growth form	areolate-rimose	granular-areolate	granular-areolate	granular-subareolate	areolate-reprose
Thallus color	olive-gray to gray	cream-white	green gray to yellow gray	white to pale yellow-green	variable (pale yellow-green, white or green-gray)
Prunia	absent or slightly pruinose on disc	absent	absent	present	absent
Hypothallus	blackish	indistinct or pale brown	absent or indistinct	indistinct	indistinct
Apothecia (mm diam.)	0.3–1.7	0.2–0.5	0.4–0.7	0.4–1.0	0.3–1.0
Thalline excipulum	absent from beginning	present when young	present when young	present when young	present when young
Epihymenium	yellow-brown	yellow-brown	brown	colorless	colorless, yellow-brown to olive
Paraphyses (μm)	1–1.5	2–2.5	1–2	1–1.5	2–2.5
Asci (μm)	50–60 × 13–21	35–45 × 10–15	32–45 × 11–15	35–45 × 10–17	30–47 × 8–12*
Ascospores (μm)	11–18 × 4–7	12–17 × 4.5–5.5	10–14 × 4–5	10–15 × 4–6	9–15.5 × 4–5 8–12 × 4–6*
Pycnoconidia (μm)	12–21 × 0.5–0.8	not observed	not observed	25 × 1.0	18–25 × 0.5–1.0
Spot test	thallus K± slightly yellow, C–, KC–	thallus K–, KC± slightly yellow	thallus K–, C+ orange, KC+ orange	thallus K+ yellow to brown, KC± yellow	thallus K–, C± orange, KC± slightly yellow
UV	negative	pale orange	bright orange	pale orange	dull orange
Substance	usnic acid, zeorin, placodiolic acid	±usnic acid, ±zeorin	usnic acid, ±zeorin, thiophanic acid, ±arthothelin	usnic acid, zeorin	usnic acid, zeorin, arthothelin, ±thiophanic acid
Reference	BDNA-L-0001218 (paratype), BDNA-L-0001220 (holotype), and BDNA-L-0001235 (paratype)	Smith et al. 2009	Nash III et al. 2004; Smith et al. 2009	Brodo et al. 2001; Smith et al. 2009	Brodo et al. 2001; Nash III et al. 2004; Smith et al. 2009; BDNA-L-0000547, BDNA-L-0000548, and BDNA-L-0000551

The morphological and chemical characteristics for several species close to the new species are referenced mainly from the previous literature. All information on the new species is measured from type specimens (BDNA-L-0001218, BDNA-L-0001220, and BDNA-L-0001235) in this study. Particularly the asci of the closest species, *Lecanorasymmicta*, was not described from the previous literature and the asci and the ascospores for the species are measured from selected specimens (BDNA-L-0000547, BDNA-L-0000548, and BDNA-L-0000551) in this study, represented with asterisk marks(*).

##### Distribution and ecology.

The species occurs on the bark of *Ligustrumobtusifolium*, *Maackiafauriei*, and *Malussieboldii*. The species is currently known from the type collections.

##### Etymology.

The species epithet indicates the lichen’s morphological similarity to the close species *Lecanorasymmicta*.

##### Notes.

The new species is morphologically similar to *Lecanorasymmicta* in its areolate and gray thallus, yellowish apothecia without developed thalline excipulum, yellowish brown epihymenium filled with pigmented granules which dissolving in K, and the presence of conidia. However, the new species differs from *L.symmicta* by its areolate-rimose thallus, blackish hypothallus, larger apothecia, absence of thalline excipulum from the beginning, narrower paraphyses, larger asci, larger ascospores, smaller pycnoconidia, chemical reaction, and the presence of placodiolic acid (Brodo et al. 2001; Nash III et al. 2004; Smith et al. 2009).

The new species is comparable to *Lecanoraaitema* (Ach.) Hepp, *L.confusa*, and *L.strobilina* in the *L.symmicta* group as all those are corticolous without soredia or leprose thallus. However, the new species differs from *L.aitema* by olive-gray to gray thallus, blackish hypothallus, larger and paler apothecia, absence of thalline excipulum from the beginning, larger asci, wider ascospores, chemical reaction, presence of placodiolic acid, and the substrate preference to deciduous trees/shrubs (vs. conifers) (Smith et al. 2009).

The new species is different from *Lecanoraconfusa* by the absence of thalline excipulum from the beginning, larger asci, larger ascospores, chemical reaction, and the presence of placodiolic acid (Nash III et al. 2004; Smith et al. 2009).

The new species is distinguished from *Lecanorastrobilina* by olive-gray to gray thallus without pruina, presence of black hypothallus, absence of thalline excipulum from the beginning, yellow-brown epihymenium, absence of crystals in apothecial section, larger asci, larger ascospores, smaller pycnoconidia, chemical reaction, and the presence of placodiolic acid (Brodo et al. 2001; Smith et al. 2009). Molecular phylogeny strongly supports that the new species is distinct in the *L.symmicta* group without any species close to it, illustrating the compared species above are located in different clades far from the new species (Figs 2 and 3). Reference Table 2 provides the key characteristics distinguishing *L.parasymmicta* from the closely related species in the *L.symmicta* group above.

All above compared species do not contain placodiolic acid and *Lecanora* species with placodiolic acid, such as *L.placodiolica* Lumbsch & Elix, *L.cinereofusca* H. Magn., *L.sarcopidoides* (A. Massal.) Hedl., *L.subravida* Nyl., *L.semitensis* (Tuck.) Zahlbr. and *L.opiniconensis* Brodo, are considered for discriminating the new species. *Lecanoraplacodiolica* differs from the new species by yellowish thallus, absence of hypothallus, presence of thalline excipulum, and darker (red-brown) discs (Lumbsch and Elix 1998). *Lecanoracinereofusca* belongs to the *L.subfusca* group with large crystals, and *L.sarcopidoides* and *L.subravida* are the members of the *L.saligna* group with presence of thalline excipulum and smaller ascospores (Van den Boom and Brand 2008). They are quite different from the new species in morphology although they produce placodiolic acid. *Lecanorasemitensis* differs from the new species by yellowish thallus, darker (dark grayish brown to yellow) discs, presence of thalline excipulum, smaller ascospores (8–12 × 4–5 μm), and the substrate preference to rock other than bark of trees (Nash III et al. 2004). *Lecanoraopiniconensis* represents yellowish thallus composed of lobate areoles, absence of hypothallus, presence of thalline excipulum, absence of zeorin, and the substrate preference to siliceous rock other than bark of trees (Brodo 1986).

##### *Lecanorasymmicta* specimens examined.

South Korea, Gangwon Province, Gangneung, Seongsan-myeon, Eoheul-ri, a forested wetland, 37°43.61'N, 128°48.13'E, 212 m alt., on bark of *Alnussibirica* Fisch. ex Turcz., 02 June 2020, B.G.Lee & H.J.Lee 2020-000347, with *Lecanorastrobilina*, *Lecidellaeuphorea* (Flörke) Kremp., *Traponoravarians* (Ach.) J. Kalb & Kalb (BDNA-L-0000547; GenBank MW832788 for ITS); same locality, on bark of *Alnussibirica*, 02 June 2020, B.G.Lee & H.J.Lee 2020-000348, two variants (one with pale brown discs and the other with yellow discs) of *Lecanorasymmicta* with *Lecidellaeuphorea*, *Rinodina* sp., *Traponoravarians* (BDNA-L-0000548; GenBank MW832789 for ITS of the former variant and GenBank MW832790 for ITS of the latter); same locality, on bark of *Alnussibirica*, 02 June 2020, B.G.Lee & H.J.Lee 2020-000351, two above variants of *Lecanorasymmicta* with *Traponoravarians* (BDNA-L-0000551; GenBank MW832791 for ITS); Pyeongchang-gun, Daegwallyeong-myeon, Hoenggye-ri, a forested wetland, 37°46.00'N, 128°42.33'E, 1,047 m alt., on bark of *Maackiaamurensis* Rupr. & Maxim., 03 June 2020, B.G.Lee & H.J.Lee 2020-000442, with *Buelliadisciformis* (Fr.) Mudd, *Buellia* sp., *Catillarianigroclavata* (Nyl.) J. Steiner, *Lecanoramegalocheila*, *Lecidellaeuphorea*, Rimulariacf.caeca, *Rinodina* sp. (BDNA-L-0000642; GenBank MW832792for ITS).

#### Protoparmeliopsis crystalliniformis

Taxon classificationFungiLecanoralesLecanoraceae

B.G. Lee & J-.S. Hur sp. nov.

5EE7451B-E6BE-5937-8A82-EAD240ADDC78

839183

Fig. 7

##### Diagnosis.

*Protoparmeliopsiscrystalliniformis* differs from *P.ertzii* by thallus color (grayish white to white vs. pale beige to ochraceous), flat to concave disc (vs. flat to convex disc), paler disc color (pale brown to dark brown vs. deep reddish brown), longer ascospores (8.5–17 × 4.2–7 μm vs. 9.4–11.3 × 5.3–6.6 μm), chemistry (thallus K+ yellow, and the presence of atranorin and rhizocarpic acid vs. all spot tests negative and no substance), and the substrate preference (sandstone or basalt vs. exposed lava).

##### Type.

South Korea, South Jeolla Province, Sinan, Ja-Eun Island, a wetland just nearby coast, 34°55.96'N, 126°04.30'E, 5 m alt., on rock (sandstone), 16 April 2020, B.G.Lee & D.Y.Kim 2020-000149, with *Ramalinayasudae* Räsänen, *Xanthoparmeliacoreana* (Gyeln.) Hale (holotype: BDNA-L-0000349; GenBank MW832797 for ITS, MW832803 for mtSSU, and MW832822 for LSU); same locality, on rock (sandstone, not calcareous), 16 April 2020, B.G.Lee & D.Y.Kim 2020-000151, with *Buelliaspuria* (Schaer.) Anzi, *Ramalinayasudae*, *Xanthoparmeliacoreana* (paratype: BDNA-L-0000351).

##### Description.

Thallus saxicolous, areolate to squamulose, linearly or web-like dispersed following furrows of substrate, not forming a rosette, pale grayish white to white, margin indeterminate, not pruinose, 100–450 μm thick; cortex pale brown, 10–20 μm thick; medulla below algal layer, 30–50 μm (sometimes 150–200 μm) thick; algal layer 50–80 μm thick, small crystals in cortex or between algal cells, dissolving but remaining in K; photobiont coccoid, cells globose to ellipsoid, 5–15 μm. Hypothallus absent.

Apothecia abundant, rounded, often contiguous or even coalescent, emerging on the surface of thallus and sessile when mature, constricted at the base, 0.3–1.7 mm diam. Disc flat or slightly concave, crenulate or entire, smooth or becoming rugose by apothecia adjoining, not pruinose, pale brown to dark brown from the beginning, 250–350 μm thick; lecanorine. Thalline excipulum persistent or rarely excluded, concolorous to thallus, 125–160 μm laterally, 80–150 μm at periphery, cortex inconspicuous, concolorous to epihymenium or slightly paler, up to 5 μm, with small and large crystals, small crystals dissolving but remaining in K, large crystals not dissolving in K. Proper excipulum inconspicuous. Epihymenium brown to pale brown, with tiny granules, granules and pigments dissolving in K, 10–20 μm high. Hymenium hyaline, 80–100μm high. Subhymenium hyaline, 30–50 μm high. Hypothecium hyaline, prosoplectenchymatous (irregular), 100–150 μm high. Oil droplets present in hymenium to upper hypothecium. Paraphyses septate, anastomosing, 1–1.5 μm wide, generally simple or occasionally branched at tips, tips not swollen or slightly swollen, not pigmented, 1.5–2 μm wide. Asci narrowly clavate, 8-spored, 40–65 × 10–12 μm (n = 6). Ascospores simple and often biguttulate in the beginning then having an oval-shaped oil drop by assembly of guttules when mature, ellipsoid to narrowly ellipsoid, rarely globose, 8.5–17 × 4.2–7 μm (mean = 11.8 × 5.5 μm; SD = 1.9(L), 0.6(W); L/W ratio 1.4–3.1, ratio mean = 2.2, ratio SD = 0.4; n = 102). Pycnidia not detected.

**Figure 7.**
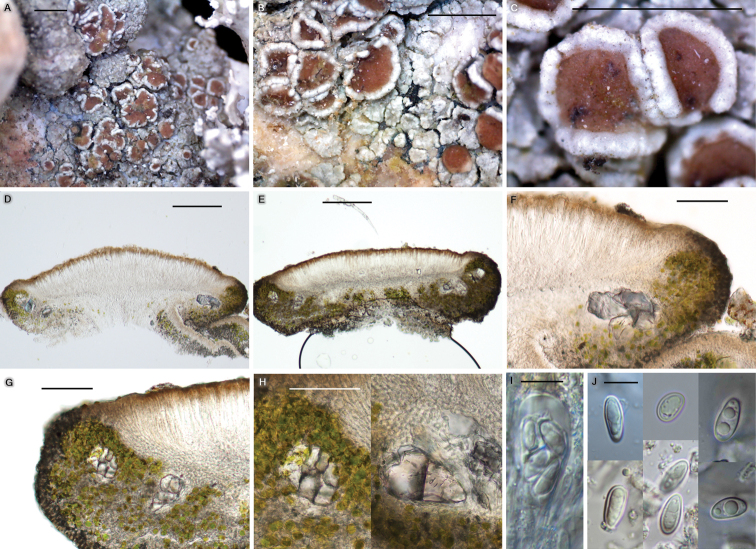
*Protoparmeliopsiscrystalliniformis* morphology (BDNA-L-0000349, holotype) **A–C** habitus and apothecia, areolate to squamulose thallus in white to whitish gray color **D–E** apothecia in vertical section **F–G** well-developed thalline margin **H** large crystals present in the thalline margin, not dissolving in KOH **I** clavate ascus **J** ascospores constantly simple and ellipsoid, often biguttulate in the beginning. Scale bars: 1 mm (**A–C**); 200 μm (**D–E**); 100 μm (**F–G**); 50 μm (**H**); 10 μm (**I–J**).

##### Chemistry.

Thallus K+ yellow, KC–, C–, Pd–. Hymenium I+ blue. UV–. Atranorin and rhizocarpic acid were detected by TLC.

**Table 3.** Comparison of the new species with close species in the genus *Protoparmeliopsis*.

Species	*P.crystalliniformis*	*P.bipruinosa*	*P.ertzii*	*P.nashii*
Thallus color	gray-white to white	pale yellow-green to gray-green-yellow, finally pale brown	pale beige to ochraceous	various shades of yellow to orange-brown cast
pruina	not present	pruina on both thallus and disc	not present	not on thallus, but present on disc
Disc evenness	flat to slightly concave	flat to slightly convex	flat to convex	flat to slightly concave
Disc color	pale brown to dark brown	yellow-brown to pale orange or green	deep reddish brown	weakly yellow to strongly yellow
Crystals	large crystals, insoluble	not observed	large crystals, insoluble	not observed
Ascospores (μm)	8.5–17 × 4.2–7	10–14 × 4–7.5	8.8–12.7 × 4.9–6.9	8–14 × 4–9
Spot test	thallus K+ yellow, KC–, C–, Pd–	thallus K–, C–; cortex KC+ yellow, P–; medulla KC–, P+ yellow or P–	all negative	thallus K– or occasionally K+, C–; cortex KC+ yellow, P–; medulla KC–, P+ yellow or less often P–
Substance	atranorin,rhizocarpic acid	usnic acid, psoromic acid, or fatty acids	no substance	usnic acid, psoromic acid, or fatty acids
Substrate	sandstone or basalt on seashore	volcanic tuff, basalt, rhyolite, or sedimentary rocks from desert scrub to woodlands	exposed lava on island	siliceous rocks (conglomerate to volcanic rocks), rarely on limestone in woodlands, desert scrub or grassland
Reference	BDNA-L-0000298, BDNA-L-0000349 (holotype), and BDNA-L-0000351 (paratype)	Nash III et al. 2004	Bungartz et al. 2020	Nash III et al. 2004

The morphological, chemical and ecological characteristics for several species close to the new species are referenced mainly from the previous literature. All information on the new species is measured from selected specimens (BDNA-L-0000298, BDNA-L-0000349, and BDNA-L-0000351) in this study.

##### Distribution and ecology.

The species occurs on the rock (sandstone or basalt) nearby coast. The species is currently known from two localities in the southern coast of South Korea.

##### Etymology.

The species epithet indicates the insoluble large crystals present in the thalline excipulum of the lichen.

##### Notes.

The new species is morphologically similar to *Protoparmeliopsisertzii* in having insoluble large crystals in the thalline excipulum and the absence of usnic acid, which are the key characteristics distinguishing them from all other species in the genus *Protoparmeliopsis*. However, the new species differs from *P.ertzii* by whitish thallus, flat to concave disc, paler disc color, longer ascospores, chemical reaction, presence of atranorin and rhizocarpic acid, and the substrate preference (Bungartz et al. 2020).

The new species is compared with *P.bipruinosa* and *P.nashii* as those are closest to the new species in molecular results (Figs 4 and 5). However, the new species differs from *P.bipruinosa* by whitish thallus, absence of pruina, presence of large crystals, and the presence of atranorin and rhizocarpic acid (Nash III et al. 2004).

The new species is different from *P.nashii* by whitish thallus, absence of pruina, presence of large crystals, and the presence of atranorin and rhizocarpic acid (Nash III et al. 2004). Reference Table 3 provides specific characteristics distinguishing *P.parasymmicta* from closely related species above in *Protoparmeliopsis*.

##### Additional specimens examined.

South Korea, South Jeolla Province, Goheung, Yeongnam-myeon, Ucheon-ri, a coastal area, 34°37.02'N, 127°29.82'E, 31 m alt., on rock (basalt), 14 April 2020, B.G.Lee 2020-000098, with *Caloplacabogilana* Y. Joshi & Hur, *Circinariacaesiocinerea* (Nyl. *ex* Malbr.) A. Nordin, Savić & Tibell, *Pertusariaflavicans* Lamy (BDNA-L-0000298; GenBank MW832796 for ITS, MW832802 for mtSSU, and MW832821 for LSU); same locality, on rock (basalt), 14 April 2020, B.G.Lee 2020-000099, with *Buellia* sp., *Circinariacaesiocinerea* (BDNA-L-0000299); same locality, on rock (basalt), 14 April 2020, B.G.Lee 2020-000100, with Buelliaaff.nashii (BDNA-L-0000300); same locality, on rock (basalt), 14 April 2020, B.G.Lee 2020-000102, with *Buellia* sp., *Caloplacabogilana*, *Circinariacaesiocinerea*, *Endocarponmaritimum* Y. Joshi & Hur, *Parmotremagrayanum* (Hue) Hale (BDNA-L-0000302); same locality, on rock (basalt), 14 April 2020, B.G.Lee 2020-000103, with *Circinariacaesiocinerea*, *Endocarponmaritimum*, *Pertusariaflavicans* (BDNA-L-0000303); same locality, on rock (basalt), 14 April 2020, B.G.Lee 2020-000105, with Buelliaaff.nashii, *Circinariacaesiocinerea*, *Pertusariaflavicans* (BDNA-L-0000305); same locality, on rock (basalt), 14 April 2020, B.G.Lee 2020-000107, with *Xanthoparmeliamexicana* (Gyeln.) Hale (BDNA-L-0000307); same locality, on rock (basalt), 14 April 2020, B.G.Lee 2020-000108, with *Caloplacabogilana*, *Endocarponmaritimum*, *Pertusariaflavicans* (BDNA-L-0000308); same locality, on rock (basalt), 14 April 2020, B.G.Lee 2020-000110, with Buelliaaff.nashii, *Buellia* sp., *Lecanoraoreinoides* (Körb.) Hertel & Rambold (BDNA-L-0000310).

### Key to *Protoparmeliopsis* and *Sedelnikovaea* species in Korea (6 taxa)

**Table:** 

1	Thalline margin with large crystals, containing atranorin and rhizocarpic acid	***P.crystalliniformis***
–	Thalline margin without large crystals	**2**
2	Thallus whitish	**3**
–	Thallus yellowish, brownish, or greenish	**4**
3	Apothecia 0.4–0.7 mm diam., disc with white pruina, epihymenium brownish, hymenium 30–40 μm high, hypothecium 70–100 μm high, ascospores wider 6–7 μm	***P.chejuensis***
–	Apothecia 0.5–1.5 mm diam., disc without pruina but thalline margin with pruina, epihymenium dull yellowish, hymenium 45–55 μm high, hypothecium 60–70 μm high, ascospores narrower 4.5–5.5 μm	***P.kopachevskae***
4	Soralia developed on thallus, apothecia absent	***P.zerovii***
–	Soralia absent, apothecia present	**5**
5	Thallus greenish gray, disc light yellow to pale brown, ascospores 8–13 × 4.5–7 μm, medulla KC– (not containing gyrophoric acid)	***P.muralis***
–	Thallus yellowish, green to grayish yellow, disc dull brown to dark brown, ascospores 17–21 × 5.5–6.5 μm, medulla KC+ yellow (containing gyrophoric acid)	***Sedelnikovaeapseudogyrophorica* (*P.pseudogyrophorica*)**

## Supplementary Material

XML Treatment for Lecanora parasymmicta

XML Treatment for Protoparmeliopsis crystalliniformis
